# Effects of Enzymatic Konjac Glucomannan Hydrolysates on Textural Properties, Microstructure, and Water Distribution of Grass Carp Surimi Gels

**DOI:** 10.3390/foods11050750

**Published:** 2022-03-04

**Authors:** Wenjin Wu, Feng Que, Xuehong Li, Liu Shi, Wei Deng, Xiaoyan Fu, Guangquan Xiong, Jing Sun, Lan Wang, Shanbai Xiong

**Affiliations:** 1 College of Food Science and Technology, Huazhong Agricultural University, Wuhan 430070, China; wuwenjin@hbaas.com; 2 Institute for Farm Products Processing and Nuclear-Agricultural Technology, Hubei Academy of Agricultural Science, Wuhan 430064, China; quefeng98@163.com (F.Q.); lxhong199712@163.com (X.L.); shiliu_hzau@163.com (L.S.); xiongguangquan@163.com (G.X.); sammi8866@sina.com (J.S.); 3 Key Laboratory of Fermentation Engineering (Ministry of Education), School of Food and Biological Engineering, Hubei University of Technology, Wuhan 430064, China; 4 College of Food & Biology Science and Technology, Wuhan Institute of Design and Sciences, Wuhan 430205, China; dengwei99098@163.com (W.D.); fuxiaoyan001@163.com (X.F.)

**Keywords:** konjac glucomannan, enzymatic hydrolysates, surimi gel, microstructure, water distribution

## Abstract

This present work investigated the influence of konjac glucomannan (KGM) enzymatic hydrolysates on the textural properties, microstructure, and water distribution of surimi gel from grass carp (*Ctenopharyngodon idellus*). The molecular weight (M_w_) of KGM enzymatic hydrolyzed by β-dextranase degraded from 149.03 kDa to 36.84 kDa with increasing enzymatic time. In the microstructure of surimi gels, KGM enzymatic hydrolysates with higher M_w_ showed entangled rigid-chains, while KGM enzymatic hydrolysates with lower M_w_ (36.84 kDa) exhibited swelled fragments. The hardness of surimi gel with a decline in KGM M_w_ exhibited first increasing then decreasing trends, while the whiteness of surimi gel increased. When KGM M_w_ decreased, the immobile water percentage of total signals decreased from 96.7% to 93.6%, and mobile water increased from 3.03% to 6.37%. In particular, the surimi gel with the addition of K2 showed better gel strength and water distributions. KGM enzymatic hydrolysates are expected to be used as a low-calorie healthy gel enhancer in surimi processing.

## 1. Introduction

Surimi, mainly composed of myofibrillar protein, is an aquatic semi-manufactured products with high protein content and low-fat content. In order to maintain elastically and plasticized properties, surimi is generally frozen in storage before processing [1]. High amounts of sucrose and sorbitol are usually added as antifreeze agents, which leads to high sugar content and calories in the product, weakening the health properties of surimi products and reducing consumer acceptance. Therefore, in the past decades, a significant body of literature focused on polysaccharides applied in frozen aquatic foods. Trehalose, kappa-carrageenan oligosaccharides, xylooligosaccharides, and alginate oligosaccharides have been reported applied on peeled shrimp to delay freezing loss [2,3,4]. Chitooligosaccharides, oligo-glucomannan, β-glucan, dextran, and chitin hydrolysate have been reported to be applied on fish protein to inhibit protein denaturation [5,6,7,8]. Konjac glucomannan as a natural polysaccharide is also used in surimi to improve surimi properties.

KGM has high molecular weight (200–2000 KDa) and is an insoluble dietary fiber with high water levels absorbing up to 60–80 times its own weight [9]. However, the attribute of high water absorption also makes KGM difficult to disperse in aqueous solutions [10]. Different modification methods can improve the solubility and dispersibility of KGM and can be better applied in surimi products. Ji, Xue, Zhang, Li, and Xue [11] reported the influence of microwave heating on the formation of KGM-myofibril networks and assumed that the expansion of KGM long-chain molecules could inhibit the aggregation of protein molecules through the steric effect. Silver crap myosin was glycated with konjac oligoglucomannan (KOG) under specific conditions, which changed the structure of myosin amide I, II, and III bonds and improved myosin solubility and heat stability [12]. Zhang, Li, Wang, Xue, and Xue [13] indicated the structure of surimi gels containing KGM largely driven by electrostatic attraction, followed by hydrogen bonding and hydrophobic interactions. Deacetylated KGM could maintain random coil structure in the myofibrillar protein and protect myosin heavy chains and actin from high temperature damage. Some researchers showed that the addition of KGM in low quality surimi could effectively improve gel properties [14,15], while the molecular characteristics of KGM played an important role in the interactions between KGM and proteins. The natural KGM, 10 kGy and 20 kGy irradiated dry KGM power, had a negative effect on the gel properties and whiteness of tilapia myofibrillar protein with low concentrations. The modification of KGM by enzymatic could improve solubility and dispersing properties [16]. Enzymatic KGM could delay the denaturation of myofibrillar protein and reduce the sweetness of *Aspitrigla cuculus* surimi products during frozen storage [17]. Enzymatic KGM also exhibited cryoprotective effects on the myofibrillar protein in our previous work [18], but the addition of enzymatic KGM on the properties of surimi gel is still unknown.

The present work aimed to investigate the effect of molecular weights of KGM enzymatic hydrolysates on the microstructure and textural properties of grass carp surimi to find the relationship among molecular characteristics of KGM enzymatic hydrolysates, water distribution, and textural properties of surimi. This work contributes to the application of KGM hydrolysate in food processing and improves the application of KGM in the cryopreservation of surimi. It provides theoretical guidance for the industrial application of KGM.

## 2. Materials and Methods

### 2.1. Materials

*Ctenopharyngodon idellus* is a widely cultivated edible freshwater fish in China, which is not an endangered or specially protected species. All methods used in this study were conducted in accordance with the guidelines of the Chinese Laboratory Animal Use and Care Legislation.

Fresh grass carp (*Ctenopharyngodon idellus*) was purchased from the Hubei Academy of Agricultural Science market (Wuhan, Hubei, China) and then transported to the laboratory alive in clean oxygenated water. Fish were exposed and immersed in ice/water slurry (temperature = 1 ± 1 °C) until no opercular movements were observed. Then, the sacrificed fish were decapitated, scaled, viscera, sliced, and washed. KGM was purchased from Hubei Konson Konjac Co., Ltd. (Ezhou, Hubei, China). β-dextranase (50,000 U/g) was purchased from Shanghai Yuanye Biotechnology Co., Ltd. (Shanghai, China). All other chemical reagents were of analytical grade.

### 2.2. Preparation of KGM Enzymatic Hydrolysates

KGM enzymatic hydrolysates were prepared according to our previous work [8]. Briefly, the pH of KGM solution was adjusted to 5.5 with 1 mol/L hydrochloric acid, and then a total of 30 mg β-glucanase powder (200,000 U/g) was added and incubated at 50 °C. The enzymatic hydrolysis times were set as 90, 120, 150, and 300 min, and the corresponding hydrolysates were named as K1, K2, K3, and K4, respectively. Upon the end of hydrolysis times, the reaction was terminated in boiling water bath for 5 min. All samples were lyophilized (10 Pa, −60 °C, 72 h) before grinding, then vacuum-packed and stored at room temperature. Lyophilized KGM was used for subsequent analysis to determine molecular weight, basic properties, and gel preparation.

### 2.3. Preparation of Surimi with KGM Enzymatic Hydrolysates

**Figure 1:**
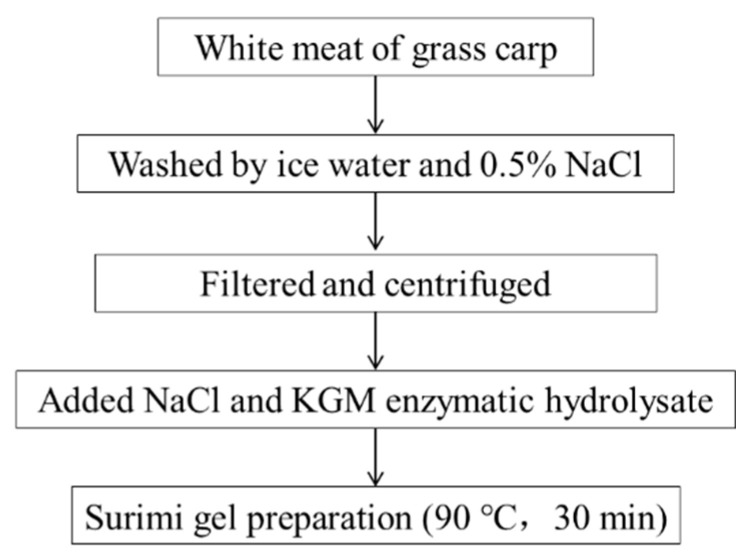
Experimental design of surimi gel with KGM hydrolysate.

Grass carp surimi was prepared according to the following methods. The white meat of grass carp was collected manually and washed successively by ice water and 0.5% NaCl solution. Subsequently, minced meat was filtered and centrifuged at 3000 r/min for 10 min to remove excess water. The moisture content of surimi was around 72%. The minced meat was chopped for 1 min, and NaCl and KGM enzymatic hydrolate solutions were added; chopping was continued for 5 min in a food processor (FP3010, De’Longhi Group, Hungary) to obtain a homogeneous mixture. The additions of NaCl and KGM enzymatic hydrolates were 2% and 1% of the entire weight, and the moisture content of surimi was adjusted to 78% with ice water. The temperature was controlled below 10 °C during surimi preparation. Surimi sol was heated at 90 °C in a water bath for 30 min and then immediately cooled with ice water. The surimi gels were stored at 4 °C for 24 h for subsequent analysis (Figure 1). The surimi gel without KGM was the control (C). The surimi gels with different enzymatic molecular weight KGM were named SK1, SK2, SK3, and SK4, respectively.

### 2.4. Molecular Weight of KGM Enzymatic Hydrolysates

The average molecular weight of KGM enzymatic hydrolysates was measured by high-performance liquid chromatography (e2695, Waters, Milford, MA, USA) with two size-exclusion chromatography columns (OHpak SB-805 HQL509108, Shodex, Japan) in series connection equipped with a refractive index detector (RID, 2414, Waters, Milford, MA, USA) and multiangle laser light scattering instrument (DAWN HELEOS II, Wyatt Technology Corporation, Goleta, CA, USA). KGM enzymatic hydrolysates (5 g) were dissolved in 400 mL 0.1 mol/L NaCl with 0.002% NaN_3_ solutions at 45 °C overnight and then filtered using a 0.45 μm nylon filter membrane before injection. The conditions were set as follows: injection volume 200 μL, flow rate 0.4 mL/min, and temperature 25 °C. An amount of 0.1 mol/L NaCl with 0.002% NaN_3_ was used as the flow phase, and dn/dc was 0.140 mL/g.

### 2.5. Intrinsic Viscosity

The intrinsic viscosities of KGM with different molecule weights were measured according to the method of Li and Xie [19]. The inner diameter of the Ubbelohde viscometer (Shanghai Gaozi Precision Instrument Co., Ltd., Shanghai, China) was 0.57 μm, and the solvent measured 0.2 mol/L NaCl.

### 2.6. Light Microcopy Analysis

Double-staining surimi gels were performed according to the method of Chiang, Li and Chen [20] with a slight modification. Samples were fixed in 4% (*w*/*v*) paraformaldehyde for 24 h and then dehydrated using a graded mixture of ethanol and xylene for 3 h each in an autotechnicon. After paraffin embedding, the slice that was 4 μm thick was dyed with a hematoxylin-eosin solution at 4 °C for 25 min and then dyed with naphthol yellow for 5 min. The morphology and distributions of KGM in surimi gels were observed using a light microscope (Eclipse CI, Nikon, Tokyo, Japan) equipped with an image system (DS-U3, Nikon, Tokyo, Japan).

### 2.7. Scanning Electron Microscope

The surimi gels were dehydrated, fixed, and critical-point dried according to the method of [21]. Cubic surimi gels (0.5 mm^3^) were fixed in 2.5% glutaraldehyde (pH 7.2) for 24 h, then dehydrated using graded ethanol, and ethanol was replaced by amyl acetate for 0.5 h. The specimens in amyl acetate were dried by liquid carbon dioxide. The surface of samples was fixed on a plate, coated with gold, and observed with scanning electron microscopy (SU8010, Hitachi, Tokyo, Japan) at 10 kV during scanning.

### 2.8. Fourier Transform Infrared Reflection (FT-IR) Spectrum

The FT-IR spectra of surimi gels were collected at room temperature by an ATR-FTIR spectrometer (VERTEX70, Bruker Optice Inc., Carlsruhe Karlsruhe, Germany). The samples were scanned 64 times from 400 to 4000 cm^−1^ with a resolution of 4 cm^−1^. The baseline was corrected by subtracting the absorption of empty ATR spectrum. The ratios of secondary structure in surimi gel were calculated from the amide III spectrum in the mid-IR spectra range from 1220 to 1330 cm^−1^ [22]. The Gaussian deconvolution, second-order derivation, and curve fitting of the amide III bond were analyzed by Peakfit 4.12 software (SeaSolve, Software Inc., Beaverton, OH, USA, 1999–2003).

### 2.9. Texture Analysis Profile (TPA)

TPA of surimi/KGM gels was carried out according to the method of Zhang, Xiong, Bakry, Xiong, Yin, Zhang, Huang, Liu, and Huang [5]. The chilling gels were taken out from the freezer and equilibrated at room temperature for 2 h and then cut into pieces (diameter: 25 mm, height: 20 mm). The TPAs of surimi/KGM gels were determined by using a TA-XT2 texture analyzer (Texture Technologies Corp., Godalming, Surrey, UK) equipped with a P/36R cylinder probe, simplified TPA mode, 40% strain, 2.0 mm/s pre-test speed, 1.0 mm/s text speed, 2.0 mm/s post-test speed, and 5 g test sensitivity. Hardness, springiness, cohesiveness, chewiness, and resilience were recorded.

### 2.10. Gel Strength

Samples for compression analysis were placed in cylinders (diameter: 25 mm, height: 20 mm) and measured in triplicate. The texture of surimi gels with different KGM hydrolysates was determined in a TA.XT2 Stable Micro Systems (Texture Technologies Corp., Godalming, Surrey, UK) texture analyzer with a P/0.5s spherical probe and then returned to start test mode at 15 mm distance, 5.0 mm/s pre-test speed, 1.0 mm/s text speed, 5.0 mm/s post-test speed, and 10 g test sensitivity. Breaking force, deformation, and gel strength were recorded.

### 2.11. Whiteness

The whiteness of surimi gels was calculated from color parameters L (lightness), a (redness/greenness), and b (yellowness/blueness) measured by a CR-400 colorimeter (Konica Minolta, Osaka, Japan). Whiteness (W) was calculated using the following equation.

(1) $$W=100\times \sqrt{{\left(100-L\right)}^{2}+a^{2}+b^{2}}$$

### 2.12. Water Holding Capacity (WHC)

The WHCs of the gels were measured by the centrifugation method (Guo, Shi, Xiong, Hu, You, Huang, and Yin [23]). Samples (5 g) were weighted and packed by filter water. Gels were centrifuged at 3000× *g* for 10 min at 4 °C. WHCs (%) were calculated according to the following equation.

(2) $$\mathrm{WHC}\left(\%\right)=\frac{m_{2}-m}{m_{1}-m}\times 100\%\;$$

*m* represents the weight of the empty centrifuge tube, *m*_1_ represents the total weight of the gel + tube before centrifugation, and *m*_2_ represents the total weight of the gel + tube after centrifugation. The test was measured six times at room temperature.

### 2.13. Low Field Nuclear Magnetic Resonance (LF-NMR)

Water molecule distributions in the surimi gels were investigated according to Ullah’s method of [24]. Two grams of gel was sliced and placed in NMR glass tubes until the temperature was equilibrated to 25 °C. The relaxation time of water molecules (T_2i_) was analyzed using the Carr-Purcell-Meiboom-Gill (CPMG) sequence. The resonance frequency of LF-NMR analyzer 20–25 (Niumag Co., Ltd., Shanghai, China) was operated at 20 MHz. The transverse relaxation times and areas were determined by using MultiExp Inv Analysis software (Niumag Co., Ltd., Shanghai, China). Each sample was measured in triplicates.

### 2.14. Magnetic Resonance Imaging (MRI)

Proton density weighted images were obtained on the same nuclear magnetic resonance (NMR) analyzer by using the multiple spin-echo (MSE) sequence. The imaging parameters were as follows: time repetition: 1000 ms; time echo: 18.2 ms; slice gap: 1.0 mm; slice number: 4l; and slice width: 0.5 mm.

### 2.15. Statistical Analysis

Each test was conducted three times, and the data were expressed as mean ± standard deviation. Statistical differences were performed with one-way analysis of variance with Duncan’s multiple range test. The software used was SPSS for Windows Version 16.0, (SPSS Inc., Chicago, Illinois, USA), and the significance level was *p* < 0.05.

## 3. Results and Discussion

### 3.1. Molecular Characteristics

The molecular weight (M_w_), molecular weight/number of average molecular weight (M_w_/M_n_), and intrinsic viscosity of KGM and four KGM enzymatic hydrolysates (KEH) samples are presented in Table 1. The M_w_ of KGM samples decreased from 894.60 kDa to 36.84 kDa with prolonged hydrolyzed time. The M_w_/M_n_ of degraded KGM samples ranged from 1.06 to 1.34, which indicated a uniform Mw distribution of KEH samples. The intrinsic viscosity of degraded KGM samples decreased from 1832.00 dL/g to 36.02 dL/g. The KGM samples with higher M_w_ possessed a higher intrinsic viscosity. The Mark-Houwink equation of KEH samples was [η] = 6.529 × 10^3^ M_w_^1.2073^, differing from the reports of Picout and Ross-Murphy [25]. The Mark-Houwink-Sakurada exponent (α) is related to the shape of macromolecules and strongly associated with the rigidity and solvation ability of polymers [26]. Generally, an α value about 0.7–0.8 represents flexible chain structures in a good solvent, while an α value above 1.0 represents a rigid chain [27]. The α value of natural KGM is 0.7317 [28], which is a flexible chain structure, while the α > 1 of the KGM hydrolysate indicates that KGM changes from a flexible molecule to a rigid molecule after enzymatic hydrolysis.

### 3.2. Microstructure

**Figure 2:**
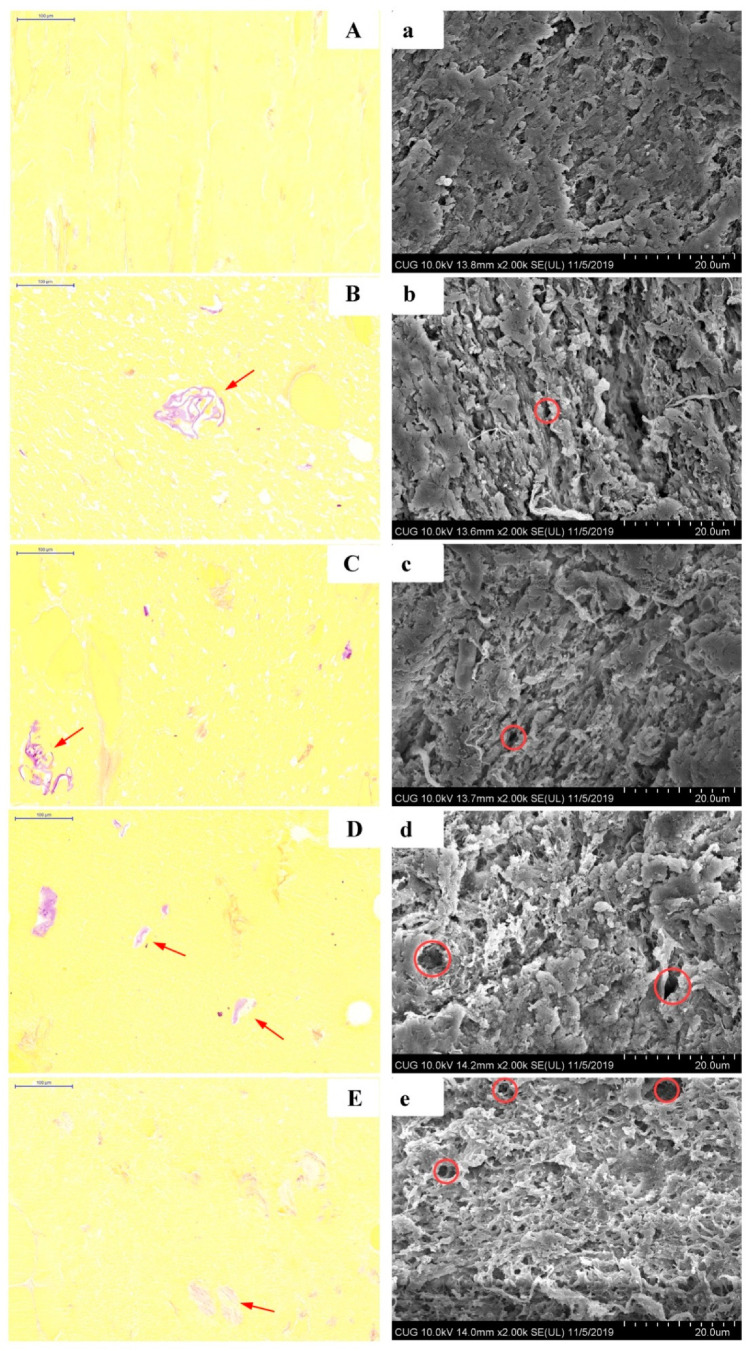
Light microscopy images (**A**–**E**) (20×) and scanning electron microscope images (**a**–**e**) (2000×) of surimi gels with different KGM enzymatic hydrolysates (KEH). (**A**–**E** and **a**–**e** represented C, SK1, SK2, SK3, and SK4, respectively).

The KEH distribution and morphology in surimi gels were observed by light microscope and SEM (Figure 2). Without KEH, the surimi gel (Figure 2A) was compact and formed a continuous bulk phase. When adding KEH, it was visualized that purple staining KGM chains and fragments were embedded in the surimi gels, similar with the observations by Yin, Yao, Ullah, Xiong, Huang, You, Hu and Shi [29] and Ullah, Hu, You, Yin, Xiong, Din, Huang, and Liu [24]. In addition, the KEH with different M_w_ exhibited different swelling states. For K2, K3, and K4, they exhibited entangled rigid chains that filled the structure of gels. Zhang, Xiong, Bakry, Xiong, Yin, Zhang, Huang, Liu, and Huang [5] observed that yeast glycan exhibited approximately circular voids in the surimi matrix. While when the KGM was further hydrolyzed (K4), KGM hydrolysates showed distributed swelled fragments surrounded with some voids in the surimi gels (SK4), which is similar to the results reported by Zhang, Li, Wang, Xue, and Xue [13].

SEM micrographs of surimi gels with and without KEH were presented in Figure 2B. In contrast to the surimi gels with KEH, the control surimi gel exhibited a compact and dense gel network. With the decrease in KGM M_w_, the microstructure of surimi gels became looser and porous. In detail, SK1 and SK2 showed lamella structure in the microstructure, while SK3 and SK4 displayed many holes in the gels. Jian, Wu, Wu, Wu, Jia, Pang, and Sun [30] thought that KGM behaved as space fillers in the gel matrix of *Tilapia* myofibrillar protein (TMP), but the microstructures in the present work were different from those in TMP, which might be due to the different preparations of gels. The degree of disorder in surimi with KEH was less than gels with okara dietary fiber [24], owing to the different solubilities of KEH and okara dietary fiber. Furthermore, the molecules of KGM tended to overlap and formed layered structure [31]. It was supposed that KEH with higher M_w_ behaved as space fillers in surimi gels, while that with lower Mw could interact with protein intermolecular hydrogen bonds and interfered with the entanglements of protein-protein molecules in surimi gels [6].

### 3.3. Secondary Structure Analysis

**Figure 3:**
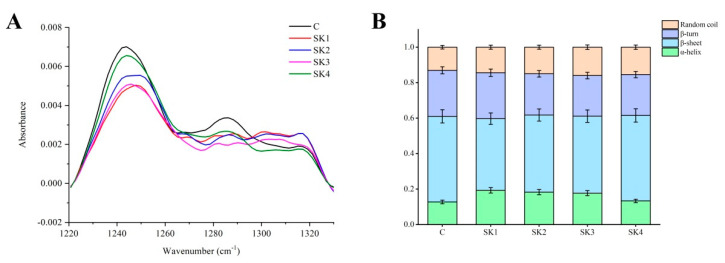
Amides III (1220–1330 cm^−1^) in FT-IR spectra (**A**) and relative amounts of secondary structure in the surimi gels ithout and with different KEH (**B**).

The infrared amide I and amide III regions are sensitive to the variations in secondary structure folding of the polypeptide and proteins. FT-IR was developed to analyze protein secondary structures by employing amides amide I (1600–1700 cm^−1^) and bond III (1220–1330 cm^−1^). However, the water molecule vibration (1640 cm^−1^) in the amide I region results in a strong interference in the accurate quantification of secondary structure [32]. In the present work, quantitative analysis of protein secondary structure in surimi gels with different KEH was measured by the amides III (1220–1330 cm^−1^) spectra (Figure 3A). In general, the amide III region consists of bond components falling in the 1330–1295 cm^−1^, 1295–1270 cm^−1^, 1270–1250 cm^−1^, and 1250–1220 cm^−1^ range, which are attributable to α-helix, β-turns, random coils, and β-sheet, respectively [33]. The relative amounts of different secondary structure were calculated from the fitted peak areas (Figure 2B). The highest bond ranging from 1250 to 1220 cm^−1^, assigned for β-sheet, was the predominant structure as an ordered network [34]. There were transformations between α-helix and β-sheet secondary structures by adding different KEH, because α-helix and β-sheets were major components related with the gelation process [35]. α-helix content increased dramatically from 12.75% (control) to 19.31% (SK1), which implicated the folding process of protein structure. In addition, the decrease in the molecular weight of KEH caused the α-helix content to decrease from 19.31% (SK1) to 13.33% (SK4), suggesting the occurrence of surimi protein unfolding. In addition, the relative amount of β-turns decreased from 25.91% to 23.02%, while that of random coil increased from 13.05% to 15.43%. α-helix was a stable secondary structure, which was susceptible to temperature, pressure, ions, pH value, and external factors [34,36,37]. This suggested that KEH enhanced the integration of protein structures during gelation, but the influence of KEH on the secondary structure weakened with M_w_ decrease.

### 3.4. Textural Properties and Whiteness

Textural properties and whiteness of surimi gels without and with KEH were revealed in Table 2 and Table 3. In comparison with the control group, the hardness, chewiness, and gel strength of surimi gels with K1 and K2 significantly increased (*p* < 0.05), while excessive degradation (K3, K4) caused the deterioration of these indicators. Among all gel samples, SK2 exhibited the lowest cohesiveness, indicating that the addition of K2 might disturb the internal bonding of the gel network. Additionally, adding KEH could observably reduce the resilience of the surimi gel (*p* < 0.05), but it has no obvious effect on springiness. There were some controversial results related to the impact of KGM on hardness and gel strength of surimi gel, which might be caused by the difference in the molecular properties of KGM and the processing technology of surimi gel [6,8,12,30]. It was found that KGM molecules with low degrees of deacetylation were partially crosslinked with myofibril and act as fillers in the gel matrix, thus improving the deformation and fracture force [38]. Combined with the results of microstructure, it was also proved that higher M_w_ KGM might act as a rigid-chain filler, resulting in an increase in hardness, chewiness, and gel strength. When M_w_ of KGM decreased, fragments of KGM hydrolylates might weaken protein aggregation or react with the myofibrillar protein [15,39], resulting in a decrease in hardness, chewiness, and gel strength.

The influence of KEH on the structure and water distribution of surimi gels was mostly depending on the molecular weight of KGM. KEH with large M_w_ tended to self-aggregate in surimi gels as being unlikely to be fully extend, while those with low M_w_ might preferred to be well extended rather than easy-to-bind water molecules or interact with proteins [40]. It was speculated that there were two possible methods to enhance the gel strength of surimi by the addition of high M_w_ KEH (K1 and K2): on the one hand, the water absorption of KEH caused the protein concentration in the matrix and, thus, led to an increase in α-helix structure in surimi protein (Figure 2); on the other hand, the aggregates of KGM hydrolysate physically filled the network of surimi (Figure 2) and improved the gel properties of surimi [41]. However, for K4 (M_w_ = 36.84 KDa), the extended chains of KEH under the steric effect might competitively react with protein molecules, thus weakening the interactions of protein-protein molecules and finally harming the surimi gel structure [5]. The self-aggregation of KEH and the interaction of KGM-protein molecules are synchronous in the surimi gel matrix, and their dominant role depends on the extension of KGM molecules.

The whiteness is one of the important criteria for evaluating the sensory quality of surimi. The whiteness of all surimi gels, ranging from 81.88 to 83.40, increased with decreasing M_w_ (Table 3). The whiteness value was influenced by the protein network [41]. The increases in whiteness might be ascribed to the rearrangement of the protein network. Additionally, KEHs were white and different from other additives such as irradiated KGM, oxidized tannic acid, egg white proteins, and yeast β-glucan [5,13,41,42].

### 3.5. WHC and Water Molecules Distribution

WHC reflects the gel network organization that intercepts the water’s ability. The influences of KEH on the WHC of surimi gels are shown in Table 3. The addition of KEH, especially K1 and K4, was detrimental to the WHC of surimi gel. The results showed that the hydrolysate of KGM interfered with the structure of protein networks and decreased the WHC of the gels, which was consistent with the hardness and chewiness of the surimi gel texture. Jian, Wu, Wu, Wu, Jia, Pang, and Sun [30] also reported that the addition of irradiated KGM powders with M_w_ 53 kDa decreased the WHC of TMP, while higher molecular weight KGM favored the WHC of TMP. Ma, Zhu, Wang, Wang, and Wang [43] thought that water competition between KGM and corn starch existed. Researchers also found that dietary fibers could envelope or destroy the myofibrillar protein and prevent water uptake [5,44]. It was suggested that the KGM with lower M_w_ involved protein-water molecules interactions by competing water molecules.

**Figure 4:**
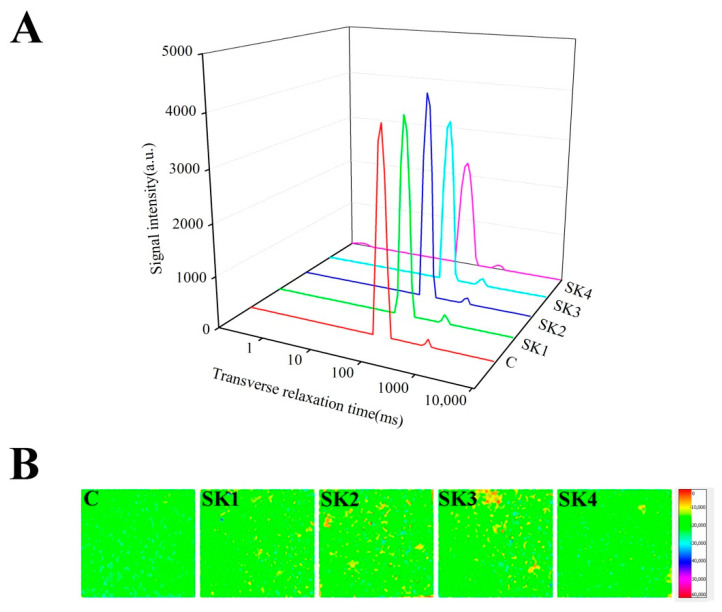
LF-NMR T_2_ relaxation (**A**) and MRI (**B**) of surimi gels with KEH.

Table 4 and Figure 4A showed the T_2_ transverse relaxation time distribution of surimi gels. LF-NMR could be used to semi-quantify the water molecules mobility and exhibit the moisture distribution by measuring relaxation characteristics of hydrogen proton in a magnetic field. The water molecules are divided into immobile and free waters by bioexponential fitting. The transverse relaxation time T_21_ ranged from 67.65 to 73.72 ms, assigned for immobile water, which was possibly located in the highly organized gel network [45]. The transverse relaxation time T_22_ ranged from 250.33 to 340.22 ms corresponded to free water that could be lost as drip water [46,47]. The major component, immobile water, accounted for 93.6–96.7% of total signals, and the other accounted for 3.03–6.37%. The range of transverse relaxation time corresponded to the previous results of surimi gels [5,24,29,48], but bound water was not observed in the present work, which might be due to the different gels and analysis sequences. The decrease in T_2_ reflected the decline in water mobility. Comparing with the control group, the addition of KEH could decrease T_21_ and T_22_, which indicated that KEH strengthened the bondage between surimi gels and immobile water. When M_w_ of KEH decreased, the progressive decline of T_22_ with the decrease in KEH’s Mw could be attributed to the interaction of KGM-water molecules in addition to the interaction of protein-water. The percentage of free water P_22_ increased with the M_w_ decrease in KGM, which meant that a part of immobile water changed to free water. The results also proved that KEH could compete with protein for water molecules. Li, Li, and Zhang [49] found that the injection of KGM gums could increase the percentage of free water of prawn. With respect to the water distribution of surimi gels, the rearrangements of water molecules caused by KEH were bidirectional: the addition of KGM hydrolysate in surimi resulted in the increase in free water (PT_22_) content [50], even though the WHC of surimi gels with KGM hydrolysate decreased (Table 3) due to the subdued mobility of free water (T_22_). The content of immobile water in surimi was reduced (PT_21_) in the presence of KEH; however, the immobile water’s mobility increased as the M_w_ of KEH decreased (T_21_), which was ascribed to the decreasing flexibility of KEH [51]. After the molecules extended, KGM might bind or react with protein molecules by hydrogen bonds, which reduced the restriction of water molecules.

MRI is a nondestructive method for monitoring water molecule distribution during food processing [52]. MRI images were used to visualize the food’s internal structure. A color bar in pseudo-color mapping was used to scale the density of protons; red color meant higher densities, while purple represents lower densities [49]. The pseudo-color mapping of the control sample was green and uniform, which indicated immobile water in surimi gels. The yellow and red spots in the image of surimi gels with KEH were observed, which meant that there was free water in surimi gels corresponding with the result of other dietary fibers in surimi gels [5,29]. With the M_w_ decrease in KEH, the SK2 sample exhibited the most yellow and red spots. Moreover, the number of yellow spots decreased with the further decline of M_w_ of KEH and the pseudo-color mapping of SK4 became even. This result was related with the dispersing degree of KEH in the surimi gel. When the M_w_ of KEH is large, the uneven dispersion of KEH could form a certain agglomeration, and the pseudo-color mapping of surimi gels exhibited a lot yellow and red spots. There was a turning point in the SK2 sample in LF-NMR T_2_ and MRI images, which might be ascribed to different specific viscosity properties of konjac gum of KEH [53].

## 4. Conclusions

The effects of KEH with different Mw on the gel properties of surimi were investigated in the present work. The results showed that KGM had two states of self-aggregation and extension in surimi gel. However, the self-aggregation of KEH and the interaction of KGM-protein molecules were synchronous in the surimi gel matrix, and the extension of KGM molecules was dominant. The entangled rigid chains displayed by high-Mw KEH were beneficial to the enhancement of surimi gel, whereas low-Mw KEH fragments hindered the enhancement of surimi gel. In particular, the surimi gel of SK2 group showed better gel strength and water distribution. Considering physicochemical and nutritional properties, the addition of K2 in surimi provided potential alternatives to the sweetness of final surimi-based products. However, the glycosylation of KEH and surimi protein requires further study.

## Figures and Tables

**Table 1:** The molecular characteristics of KEH.

Samples	M_w_ (kDa)	Polydisversity (M_w_/M_n_)	Root Mean Square Radius (nm)	Intrinsic Viscosity (dL/g)
KGM	894.60 ± 21.20 ^e^	1.06	108.10 ± 1.50 ^d^	1832.00 ± 2.29 ^e^
K1	149.03 ± 1.91 ^d^	1.14	45.20 ± 1.17 ^c^	397.75 ± 9.55 ^d^
K2	128.70 ± 5.90 ^c^	1.15	42.20 ± 1.29 ^b^	303.55 ± 5.42 ^c^
K3	118.75 ± 2.76 ^b^	1.20	42.00 ± 1.13 ^b^	188.70 ± 8.16 ^b^
K4	36.48 ± 1.23 ^a^	1.34	31.25 ± 1.12 ^a^	36.02 ± 0.67 ^a^

Note: Values in the same column with different letters were significantly different (*p* < 0.05).

**Table 2:** Texture profile analysis (TPA) of surimi gels with different KEH.

Samples	Hardness (g)	Springiness	Cohesiveness	Chewiness (g)	Resilience
C	3785.94 ± 95.63 ^c^	0.887 ± 0.024 ^a^	0.756 ± 0.010 ^a^	2539.07 ± 81.93 ^bc^	0.432 ± 0.008 ^a^
SK1	4028.11 ± 89.38 ^b^	0.873 ± 0.041 ^a^	0.754 ± 0.007 ^a^	2649.88 ± 97.90 ^b^	0.421 ± 0.008 ^b^
SK2	4348.46 ± 192.02 ^a^	0.884 ± 0.025 ^a^	0.742 ± 0.008 ^b^	2855.24 ± 174.84 ^a^	0.413 ± 0.007 ^b^
SK3	3476.50 ± 218.31 ^c^	0.900 ± 0.030 ^a^	0.752 ± 0.005 ^ab^	2348.26 ± 96.065 ^c^	0.412 ± 0.005 ^b^
SK4	2869.20 ± 182.68 ^d^	0.892 ± 0.023 ^a^	0.7528 ± 0.005 ^a^	1925.33 ± 97.83 ^d^	0.414 ± 0.007 ^b^

Note: Values in the same column with different letters were significantly different (*p* < 0.05).C: surimi gel without KGM. SK1, SK2, SK3, and SK4: surimi gels with different enzymatic molecular weight KGM.

**Table 3:** Gel strength, whiteness, and WHC of surimi gels with different KEH.

Samples	Breaking Force (g)	Deformation (cm)	Gel Strength (g.cm)	Whiteness	WHC
C	361.09 ± 16.13 ^c^	0.83 ± 0.03 ^a^	301.24 ± 26.30 ^c^	81.88 ± 0.23 ^c^	76.36 ± 0.82% ^a^
SK1	325.73 ± 13.78 ^bc^	1.08 ± 0.31 ^a^	342.40 ± 29.83 ^b^	82.41 ± 0.33 ^b^	69.54 ± 1.35% ^bc^
SK2	305.04 ± 59.16 ^ab^	1.38 ± 0.21 ^b^	412.12 ± 10.46 ^a^	82.35 ± 0.19 ^b^	72.85 ± 2.52% ^ab^
SK3	332.09 ± 6.42 ^bc^	0.81 ± 0.03 ^a^	270.10 ± 6.78 ^c^	83.02 ± 0.11 ^a^	73.07 ± 2.22% ^a^
SK4	276.46 ± 15.21 ^a^	0.80 ± 0.05 ^a^	222.01 ± 22.09 ^d^	83.40 ± 0.14 ^a^	68.40 ± 2.18% ^c^

Note: Values in the same column with different letters were significantly different (*p* < 0.05).C: surimi gel without KGM. SK1, SK2, SK3, and SK4: surimi gels with different enzymatic molecular weight KGM.

**Table 4:** Relaxation times and corresponding peak areas of surimi gels with different KEH.

Samples	T_21_ (ms)	PT_21_ (%)	T_22_ (ms)	PT_22_ (%)
C	72.71 ± 0.38 ^a^	96.97 ± 0.06 ^a^	340.22 ± 23.35 ^a^	3.03 ± 0.06 ^d^
SK1	67.65 ± 0.95 ^c^	95.08 ± 0.46 ^c^	290.42 ± 10.27 ^b^	4.92 ± 0.47 ^b^
SK2	69.22 ± 0.35 ^b^	95.83 ± 0.39 ^b^	288.37 ± 10.79 ^b^	4.17 ± 0.39 ^c^
SK3	72.44 ± 1.28 ^a^	94.46 ± 0.54 ^c^	277.42 ± 18.72 ^bc^	5.54 ± 0.53 ^b^
SK4	73.75 ± 0.70 ^a^	93.63 ± 0.16 ^d^	250.33 ± 15.15 ^c^	6.37 ± 0.16 ^a^

Note: Values in the same column with different letters were significantly different (*p* < 0.05).C: surimi gel without KGM. SK1, SK2, SK3, and SK4: surimi gels with different enzymatic molecular weight KGM.

## Data Availability

Not applicable.
